# Suppression of TGF-β pathway by pirfenidone decreases extracellular matrix deposition in ocular fibroblasts *in vitro*

**DOI:** 10.1371/journal.pone.0172592

**Published:** 2017-02-23

**Authors:** Thomas Stahnke, Bhavani S. Kowtharapu, Oliver Stachs, Klaus-Peter Schmitz, Johannes Wurm, Andreas Wree, Rudolf Friedrich Guthoff, Marina Hovakimyan

**Affiliations:** 1 Department of Ophthalmology, Rostock University Medical Center, Rostock, Germany; 2 Institute for Biomedical Engineering, Rostock University Medical Center, Rostock, Germany; 3 Department of Anatomy, Rostock University Medical Center, Rostock, Germany; Medical University of South Carolina, UNITED STATES

## Abstract

In glaucoma surgery, fibrotic processes occur, leading to impairment of liquid outflow. Activated fibroblasts are responsible for postoperative scarring. The transforming growth factor-β (TGF-β) pathway plays a key role in fibroblast function, differentiation and proliferation. The aim of this study was the characterization of the fibrotic potential of two subtypes of primary human ocular fibroblasts and the attempt to inhibit fibrotic processes specifically, without impairing cell viability. For fibrosis inhibition we focused on the small molecule pirfenidone, which has been shown to prevent pulmonary fibrosis by the decrease of the expression of TGF-β1, TGF-β2 and TGF-β3 cytokines. For *in vitro* examinations, isolated human primary fibroblasts from Tenon capsule and human intraconal orbital fat tissues were used. These fibroblast subpopulations were analyzed in terms of the expression of matrix components responsible for postoperative scarring. We concentrated on the expression of collagen I, III, VI and fibronectin. Additionally, we analyzed the expression of α-smooth muscle actin, which serves as a marker for fibrosis and indicates transformation of fibroblasts into myofibroblasts. Gene expression was analyzed by rtPCR and synthesized proteins were examined by immunofluorescence and Western blot methods. Proliferation of fibroblasts under different culture conditions was assessed using BrdU assay. TGF-β1 induced a significant increase of cell proliferation in both cell types. Also the expression of some fibrotic markers was elevated. In contrast, pirfenidone decreased cell proliferation and matrix synthesis in both fibroblast subpopulations. Pirfenidone slightly attenuated TGF-β1 induced expression of fibronectin and α-smooth muscle actin in fibroblast cultures, without impairing cell viability. To summarize, manipulation of the TGF-β signaling pathway by pirfenidone represents a specific antifibrotic approach with no toxic side effects in two human orbital fibroblast subtypes. We presume that pirfenidone is a promising candidate for the treatment of fibrosis following glaucoma surgery.

## Introduction

Glaucoma refers to a group of multifactorial optic neuropathies that have in common a progressive degeneration of retinal ganglion cells and their axons, leading to a thinning of the retinal nerve fibers comprising the optic nerve [1]. Worldwide, glaucoma is the leading cause of irreversible blindness, affecting more than 60 million people with 8.4 million people afflicted from bilateral blindness [2]. An epidemiological review has concluded that 1 in 40 adults over 40 years of age suffers from glaucoma, with disease prevalence increasing significantly with age [3,4]. The most prevalent form of glaucoma in Caucasian population is primary open angle glaucoma, characterized by impaired outflow of aqueous humor (AH) through the trabecular meshwork [5].

While the pathogenesis of glaucomatous optic neuropathy still remains an area of research, elevated intraocular pressure (IOP) is believed to be the strongest risk factor [6]. Reducing IOP and its fluctuations is so far the only proven means to slow or halt disease progression [7]. In therapeutic terms, IOP-lowering can be achieved by pharmaceutical treatment and laser application to trabecular meshwork or ciliary body structures, or microsurgical procedures [8]. The goal of these interventions is to adjust corresponding IOP at which disease progression can be halted.

Pharmaceutical treatment with topical eye drops can cause proinflammatory effects, inducing ocular discomfort and burning sensation [9], which may consequently contribute to the poor patient adherence [10]. The laser treatment is not suitable for every glaucoma case and often may need to be repeated or combined with local therapy in the long-term to keep eye pressure stable. Glaucoma filtration surgery is an alternative which is indicated when the glaucomatous optic neuropathy progresses despite medical and laser therapies [11]. Trabeculectomy remains to be the most commonly performed antiglaucomatous surgical procedure and is considered as a gold standard [12–14].

In the recent decade, glaucoma drainage devices (GDDs) have been introduced as an alternative to the glaucoma filtering surgery [15]. Studies have shown that GDD implantation can be at least as effective as trabeculectomy at reducing IOP and the need for further surgery over 5 years [16]. Like filtrating surgery, the GDDs work by creating a new outflow route allowing AH draining from anterior chamber to subconjunctival or suprachoroidal space [17].

Any surgical tissue manipulation is generally accompanied by a wound healing process, which affects the long-term success of these procedures. Particularly, scarring and fibrotic encapsulation at the surgical site can lead to surgical failure. Fibrosis is defined as an excessive tissue growth, characterized by uncontrolled fibroblast proliferation, migration, their transformation into α-smooth muscle actin (α-SMA)-producing myofibroblasts and deposition of extracellular matrix (ECM) components, including various members of collagen superfamily and fibronectin [18]. Therefore, several attempts have been made to suppress the fibroblasts activation in order to inhibit surgical scarring and fibrosis. The most commonly used antimetabolites 5-fluorouracil (5-FU) and mitomycin C (MMC) inhibit cells in a non-selective manner, are cytotoxic and usually accumulate in areas where no inhibitory effect is wanted [19,20]. As a result, antimetabolite treatment is associated with undesired side effects such as corneal endothelial damage or bleb leaking [21]. More recent strategies utilize less cytotoxic molecules which are expected to inhibit fibroblasts through manipulation of different cellular pathways. Promising results have been reported following inhibition of TGF-β (transforming growth factor-β) signal transduction pathway [22].

In the present study, we examined the antifibrotic potential of the small molecule pirfenidone (PFD), which has been used as an oral formulation for systemic treatment of idiopathic pulmonary fibrosis [23]. We hypothesized that PFD inhibits fibroblasts proliferation and expression of ECM-molecules in TGF-β-induced fibrotic cell cultures of ocular fibroblast subtypes from regions possibly used as drainage outflow areas.

## Materials and methods

### Materials

Cell culture media (D6429), flasks (TPP 25 cm^2^ and 75 cm^2^) and pirfenidone ((PFD); P2116) were purchased from Sigma-Aldrich (Taufkirchen, Germany). Cell culture dishes (10 cm) and 12 well plates were from Nunc (Thermo Fisher scientific, Massachusetts, USA), 12 mm coverslips and 96 well microtiter plates from PAA (Cölbe, Germany). Cell Proliferation ELISA, BrdU (chemiluminescence) was supplied from Roche Diagnostics (Mannheim, Germany). SDS-PAGE prestained molecular weight standard (Roti-Mark PRESTAINED, T852.1) was purchased from Carl Roth GmbH & Co. KG (Karlsruhe, Germany). The recombinant human cytokine transforming growth factor β1 ((TGF-β1); 11343160) was purchased from Immunotools (Friesoythe, Germany). All antibodies used in this study were raised against human antigen and are listed in the Table 1.

**Table 1:** Antibodies used in this study.

Reagent	Supplier	Cat. number
mouse monoclonal anti-collagen I	Abcam (Cambridge, UK)	ab90395
mouse monoclonal anti-collagen I	Abcam (Cambridge, UK)	ab6308
mouse monoclonal anti-collagen I	Santa Cruz (Dallas, USA)	sc-59772
mouse monoclonal anti-collagen VI	Abcam (Cambridge, UK)	ab78504
mouse monoclonal anti-α-SMA	Abcam (Cambridge, UK)	ab7817
rabbit polyclonal anti-α-SMA	Abcam (Cambridge, UK)	ab5694
rabbit polyclonal fibronectin	DPC Biermann GmbH (Germany)	DP013
mouse monoclonal fibronectin	Sigma-Aldrich (Germany)	F7387
mouse monoclonal anti-β-tubulin	Sigma-Aldrich (Germany)	T5293
secondary HRP-conjugated anti-rabbit IgG	BIO-RAD (Munich, Germany)	170–6515
secondary HRP-conjugated anti-mouse IgG	GE Healthcare (Amersham; Buckinghamshire, UK)	NXA931
secondary Alexa Fluor 488-conjugated donkey anti-mouse IgG (H+L)	Dianova GmbH, (Hamburg, Germany)	715-545-151
secondary Cy3-conjugated donkey anti-rabbit IgG (H+L)	Dianova GmbH, (Hamburg, Germany)	711-165-152

### Cell culture

This study was approved by the ethics committee of the University of Rostock (approval ID: A 2011 11) and followed the guidelines of the Declaration of Helsinki.

Primary cultures of human orbital fibroblasts (hOFs) were prepared from donors’ orbital fat tissues (Institute of Anatomy, Rostock University Medical Center, Germany) as described previously [24]. Briefly, after enucleation orbital fat was collected. Each tissue was cut into pieces approximately 1×1 mm in size, placed in 12 well plastic culture plates in DMEM with 50 U/ml of penicillin, 50 μg/ml streptomycin and 10% FCS, and incubated at 37°C in a humidified (95%) incubator under 5% CO2. Medium was changed three times a week. Upon reaching confluence, cells were trypsinized with 0.25% trypsin/EDTA solution in phosphate buffered saline (PBS) and subcultured in 25 cm^2^ plastic cell culture flasks.

Primary cultures of human Tenon fibroblasts (hTFs) were prepared after strabismus and enucleation surgeries (Department of Ophthalmology, Rostock University Medical Center, Germany). A written informed consent was obtained from all participants. Small pieces of non-functional episclera (Tenon tissues) were removed during surgeries. Accordingly, Tenon tissues were treated like orbital fat tissues. After Tenon fibroblasts proliferated to a confluent monolayer, cells were trypsinized and subcultured in 25 cm^2^ cell culture flasks.

After reaching a confluent layer in 25 cm^2^ culture flasks, fibroblasts were trypsinized again and seeded in 10 cm culture dishes and in 75 cm^2^ cell culture flasks, respectively. For immunofluorescence analysis cells were seeded on 12 mm plastic coverslips (PAA, Cölbe, Germany) and cultured until 60%–70% confluence was reached. For all analyses fibroblasts of passage three to five were used. The fibroblastic phenotype was confirmed by immunohistochemistry using anti-vimentin antibody to verify the mesenchymal origin of cells, and anti-FBSP (fibroblast surface protein), as a fibroblast marker (data not shown).

For stimulation- and inhibition experiments, human fibroblast subpopulations were starved for 24 h under serum-free conditions, followed by the application of TGF-β1 [10 ng/ml], PFD [10^−3^ mol/l] or the combination of TGF-β1 [10 ng/ml] and PFD [10^−3^ mol/l] for 48 h. The effective concentration of TGF-β1 was determined earlier by the induction of α-SMA expression (data not shown). The concentration of PFD was chosen based on own previous experiments, demonstrating the non-toxicity of PFD at 10^−3^ mol/l.

Untreated fibroblasts serum-starved for 72 h served as a control.

### BrdU cell proliferation assay

TGF-β1, PFD and the combination of both were also tested for their impact on fibroblast proliferation. 2000 cells were seeded into each well of a 96-well microtiter plate in growth medium and incubated under standard conditions for one day. After starving for 24 h, cells were incubated with TGF-β1 [10 ng/ml], PFD [10^−3^ mol/l] and the combination of both in culture medium with or without FCS. After a 32 h incubation period BrdU (100 μM) was added to each well and cells were allowed to grow for additional 16 h. The incorporation of BrdU into DNA was measured according to the supplier’s instructions of the Cell Proliferation ELISA, BrdU (chemiluminescence) (Roche Diagnostics, Mannheim, Germany). Cells not subjected to TGF-β1 or PFD served as a negative control (NC). All measurements were run in quadruplicate and proliferation values were calculated relative to the NC whose viability was set to 100%.

### RNA isolation and reverse transcription

For RNA isolation, cells were harvested into TRIzol (Life Technologies GmbH, Darmstadt, Germany) reagent. Briefly, after the addition of 10% chloroform (Baker, Deventer, Netherlands), cells were vortex mixed and purified RNA was isolated in two centrifugation steps. The aqueous supernatant was adjusted to 35% ethanol and loaded onto an RNeasy column (QIAGEN, Hilden, Germany). After washing the column, residual DNA was digested with DNase I (QIAGEN) treatment followed by three washing steps. Total RNA was eluted with 100 μl of sterile, RNase free water. RNA quality was assessed for size and purity on a 1.5% agarose gel.

For reverse transcription of isolated RNA into cDNA, 1 μg of the extracted total RNA was subjected to reverse transcription using random hexamer primers (First Strand cDNA Synthesis Kit, Fermentas, St. Leon-Rot, Germany). Sequences for PCR primers were generated with the Primer3 software (primer3.ut.ee/). Primers used in this study (Table 2) were purchased from Eurofins MWG-Operon (Ebersberg, Germany), which included alpha smooth muscle actin (a-SMA), fibronectin1, collagen type I alpha 2 (COL1A2), collagen type III alpha 1 (COL3A1), matrix metalloproteinase-2 (MMP2) and thrombospondin (THBS2). Glyceraldehyde-3-phosphate dehydrogenase (GAPDH) and 18S ribosomal RNA (RNA18S5) were used as internal reference.

**Table 2:** Primer sequences used in this study.

Primer	sequence [5' - 3']
aSMA	GTGTGTGACAATGGCTCTGG
	GCCAGATCTTTTCCATGTCG
Fibronectin1	AATATCTCGGTGCCATTTGC
	CGGGAATCTTCTCTGCTAGC
COL1A2	CTGGACCTCCAGGTGTAAGC
	TGGCTGAGTCTCAAGTCACG
COL3A1	AACACGCAAGGCTGTGAGACT
	GCCAACGTCCACACCAAATT
MMP2	TGATCTTGACCAGAATACCATCGA
	GGCTTGCGAGGGAAGAAGTT
THBS2	CGTGGACAATGACCTTGTTG
	GCCATCGTTGTCATACTCAG

### Quantitative PCR

Quantitative PCR reactions were performed by using equal amounts of cDNAs along with the required set of primers and PCR master mix (Thermo Scientific Finnzymes, Schwerte, Germany) containing dNTPs, Taq polymerase and SYBR green. Initially, cDNA was denaturated and Thermus aquaticus DNA polymerase was activated at 98°C for 10 min. Afterwards, 40 PCR cycles (94°C 10 s denaturation, 60°C 20 s annealing, 72°C 30 s elongation) were run in a Master Cycler realplex² (Eppendorf, Hamburg, Germany). Samples without any cDNA were also included in each amplification reaction to serve as negative controls. After the PCR, amplification products were analyzed on 1.5% agarose gels by electrophoresis. All PCR reactions were run in quadruplicate and the data were analyzed by using Graph Pad Prism 5 software. One way ANOVA was performed to determine differences between the groups, and p-values <0.05 were considered statistically significant.

### Immunofluorescence

For immunocytochemistry cells were allowed to grow on plastic cover slips (12 mm, PAA, Cölbe, Germany) until they reached an optimal density (subconfluent monolayer). After the incubation times with TGF-β1 and/or PFD the coverslips were washed with PBS and cells were fixed with 3% paraformaldehyde (PFA) for 10 min. After fixation, cells were pretreated with 0.1% Triton X-100 containing 2% FCS (30 min) to permeabilize cell membranes for intracellular protein staining, followed by incubation with the primary antibodies in PBS for 60 min. Antibodies were used at the following dilutions: rabbit polyclonal anti-fibronectin (1:100), mouse monoclonal anti-α-SMA (1:100), mouse monoclonal anti-collagen I and VI (1:100). After incubation cells were washed three times with PBS, followed by an incubation with the secondary antibodies for 45 min. Secondary antibodies were used at the following dilutions: donkey anti-mouse IgG (H+L)-Alexa Fluor 488 (1:50), or donkey anti-rabbit IgG (H+L)-Cy3 (1:100). After incubation with secondary antibodies, cells were washed again three times with PBS and mounted. Control experiments, using the secondary antibodies only, did not show unspecific staining. Nuclei were stained with 4, 6-diamidino-2-phenylindole (DAPI) (1μg/ml) included in the mounting medium (Vectashield, Vector Laboratories LTD., Peterborough, UK). Fluorescent labeling was analyzed using a Nikon confocal fluorescence microscope equipped with a digital camera (Nikon Eclipse E400 with D-Eclipse C1, Düsseldorf, Germany). All images depicted in this study were from a single plane through fibroblast cell monolayers equipped with a 40x objective using the same settings.

### Western blot analysis

For Western blot analysis, primary human fibroblast subpopulations were washed with PBS and scraped off into sample buffer containing 1% SDS. All samples were boiled for 10 minutes. After cooling on ice, protein contents in the samples were determined according to Neuhoff et al. [25]. For separation by SDS-PAGE, total cellular extracts (10–30 μg protein per lane) were loaded in 7.5% or 10% polyacrylamide gels for larger and smaller proteins, respectively. By immunoblotting proteins were transferred to PVDF membranes (0.2 μm, BIO-RAD, Munich, Germany). After blocking the membranes with 5% nonfat dry milk powder in TRIS-buffered saline (TBS) for 30 min, blots were incubated with individual primary antibodies at 4°C over night. Following antibodies were used (dilutions are given in brackets): mouse monoclonal anti-collagen I and VI (1:500), mouse monoclonal anti-fibronectin (1:1,000), mouse monoclonal anti-β-tubulin (1:1,000) and mouse monoclonal anti-α-SMA (1:500). After three washing steps with TBS including Tween 20, membranes were incubated with secondary HRP-conjugated anti-mouse (1:2,500) or anti-rabbit (1:2,500) IgG. Visualization of bound antibodies was performed by the enhanced chemiluminescence (ECL) procedure as described by the manufacturer (Thermo scientific, Pierce, Rockford, USA).

Optical density of each band was normalized to corresponding β-tubulin band. The blots were quantified using ImageJ software as per guidelines given by Gassmann et al. [26].

### Statistical analysis

Statistical analyses were performed using GraphPad Prism 5 data analysis software (GraphPad Software, La Jolla, USA). Significance tests of different culture conditions were conducted using one way ANOVA. Differences were considered as statistically significant for p-values <0.05. Bar charts were generated using means and the standard deviation (SD).

## Results

In this study we examined the antifibrotic effect of the orphan drug PFD on different human ocular fibroblast subpopulations, which are involved in postsurgical events of glaucoma treatment. In postoperative wound healing processes these fibroblasts are activated to obturate surgical wounds by migration, proliferation and production of ECM components.

### Cell morphology

**Fig 1:**
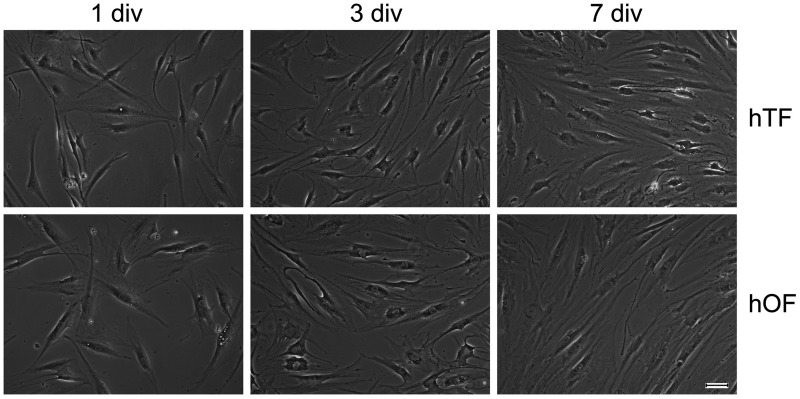
Human fibroblast subpopulations *in vitro*. Primary fibroblast subpopulations from Tenon’s capsule (hTFs) and fibroblasts from orbital fat (hOFs) were cultured for 1, 3 and 7 days, respectively. div = days *in vitro*. Bar represents 25 μm.

For our experiments, primary human fibroblasts from Tenon’s capsule (hTFs) and from the intraconal orbital fat depot (hOFs) were examined *in vitro* (Fig 1). Fibroblast subpopulations exhibited typical spindle-shaped morphology and no differences concerning size and shape of the cells could be observed between hTFs and hOFs.

### Cell proliferation

**Fig 2:**
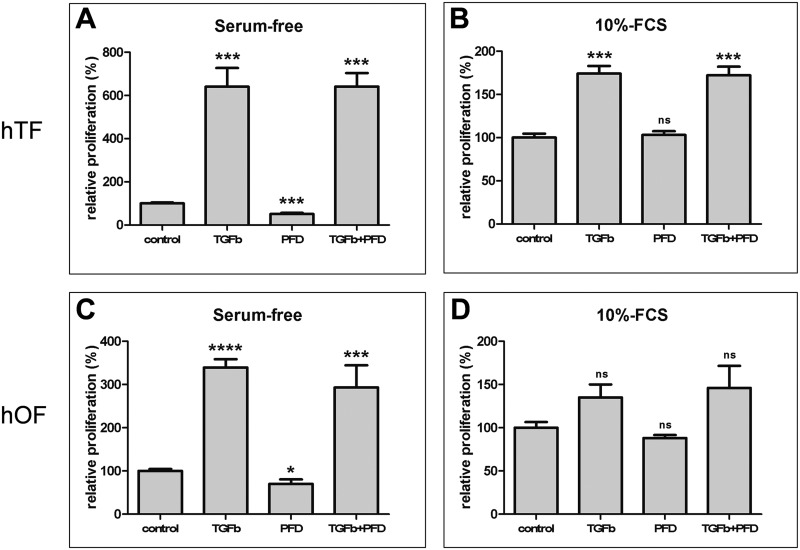
Relative proliferation of hTFs and hOFs in response to TGF-β1 and PFD *in vitro*. Fibroblasts were treated with TGF-β1 [10 ng/ml], PFD [10^−3^ mol/l] or the combination of TGF-β1 [10 ng/ml] and PFD [10^−3^ mol/l] for 48 h under serum-free (A and C) and serum (10% FCS) conditions (B and D) as indicated. NC (proliferation rate of untreated cells) was set to 100%. Data are presented as mean ± SD. The results represent the means of three independent experiments. Level of significances: *p≤0.05; **p≤0.01; ***p≤0.001; ****p≤0.0001.

The stimulation of hTFs with TGF-β1 resulted in an increase of cell proliferation to 640% of NC (Fig 2A).

In contrast, cells incubated with PFD exhibited only 51% of NC’s proliferation, though this decrease was statistically significant. When incubated with a combination of TGF-β1 and PFD in serum-free conditions, the hTFs exhibited 641% relative proliferation (Fig 2A).

Under 10% FCS conditions (Fig 2B), the proliferation rate was 174% for hTFs incubated with TGF-β1 alone and 172% for cells incubated with a combination of TGF-β1 and PFD (Fig 2B). PFD alone did not influence the cell proliferation at all (Fig 2B).

In general, similar observations were made for hOFs (Fig 2C and 2D). Alterations of proliferation rate of hOFs under serum free (Fig 2C) and 10% FCS (Fig 2D) conditions demonstrated the same trends as observed for hTFs. Following stimulation of hOFs with TGF-β1 under serum free conditions, the relative proliferation of hOFs increased to 339% of NC. In contrast PFD acted antiproliferative by significantly dropping the proliferation rate to 70% of NC (Fig 2C). The stimulatory effect of TGF-β1 was weakened in hOFs incubated with a combination of TGF-β1 and PFD; these cells exhibited 293% of relative proliferation which was less compared with that of cells with TGF-β1 alone (339%) (Fig 2C). Under 10% FCS conditions, hOFs incubated with TGF-β1 alone or its combination with PFD exhibited comparable proliferation rates (135% and 146% of NC, respectively) (Fig 2D). PFD alone led to a non-significant proliferation decrease by 12% (Fig 2D).

Given the fact that TGF-β1 induced cell stimulation was more pronounced in serum-free cell culture conditions, which was expectable as TGF-β has better access to fibroblast cell surface receptors in serum free media, all further experiments were performed in serum-free cell culture conditions.

### Gene expression patterns

**Fig 3:**
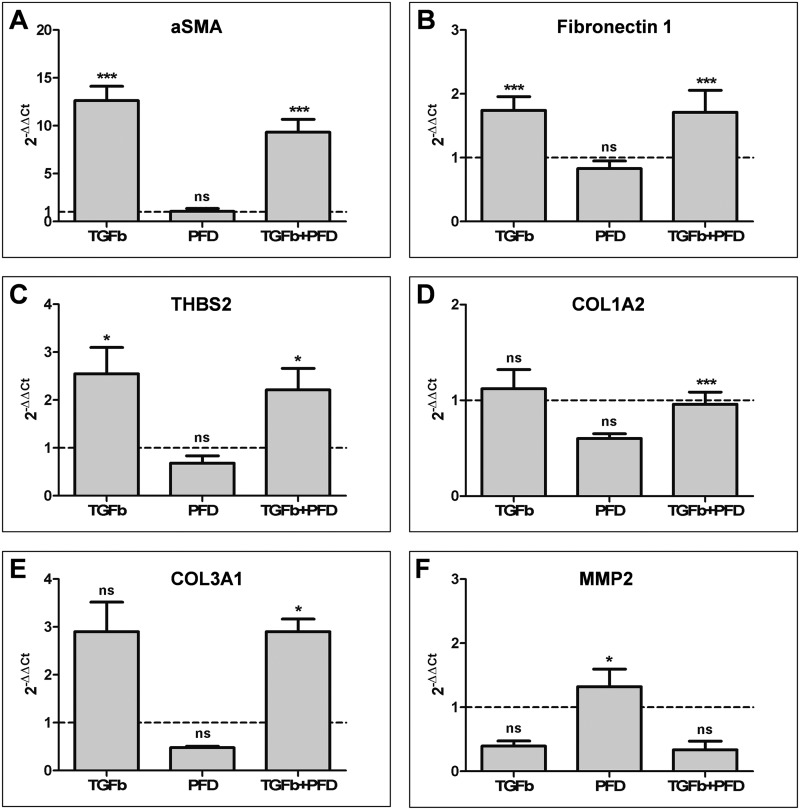
RT-PCR analysis of stimulated (TGF-β1) and suppressed (PFD) primary hTFs. Cultures of hTFs were treated with TGF-β1 [10 ng/ml], PFD [10^−3^ mol/l] or the combination of both, TGF-β1 and PFD for 48 h under serum-free culture conditions. NC (expression level of respective gene in untreated cells, dotted line) was set to 1. Data are presented as mean ± SD. The results represent the means of four independent experiments. Level of significances: *p≤0.05; **p≤0.01; ***p≤0.001. Differences between TGF-β1 and TGF-β1+PFD are statistically not significant.

In the presence of TGF-β1, a 12-fold increase in the expression of α-SMA mRNA was seen in hTF cultures, whereas there was no difference in α-SMA expression in the presence of PFD alone compared to the control cultures (Fig 3A). In hTFs cultured with a combination of TGF-β1 and PFD, a 9-fold increase could be observed, suggesting that the effect of TGF-β1 can be partly abrogated by the addition of PFD (Fig 3A). However, by comparison of TGF-β1 and combination of TGF-β1 and PFD the difference did not reach statistical significance.

In contrast, fibronectin (Fig 3B) and THBS2 (Fig 3C) mRNA expression was doubled in the presence of TGF-β1 and in the combination of TGF-β1 and PFD. It remained same to the control baseline values in the presence of PFD alone, revealing that PFD has no effect on the expression of these mRNAs (Fig 3B and 3C).

In case of COL1A2 mRNA expression, no change was noticed in the presence of TGF-β1 or its combination with PFD when compared to control values. Instead, a decrease of mRNA expression was observed after treatment with PFD alone (Fig 3D). An almost 3-fold increase in the expression of COL3A2 mRNA was observed in the presence of TGF-β1 or its combination with PFD compared to the control conditions (Fig 3E). However, treatment with PFD alone reduced COL3A2 mRNA expression when compared to the baseline values (Fig 3E). Conversely, we observed an increase in the expression of MMP2 mRNA in the presence of PFD alone, whereas the same gene was slightly downregulated in cells cultured with TGF-β1 or its combination with PFD (Fig 3F).

Notably, when comparing the expression levels of fibronectin (B), THBS2 (C), COL1A2 (D), COL3A1 (E) and MMP2 (F) between cultures treated with TGF-β1 only or TGF-β1 and PFD in combination, no statistically significant difference could be observed.

**Fig 4:**
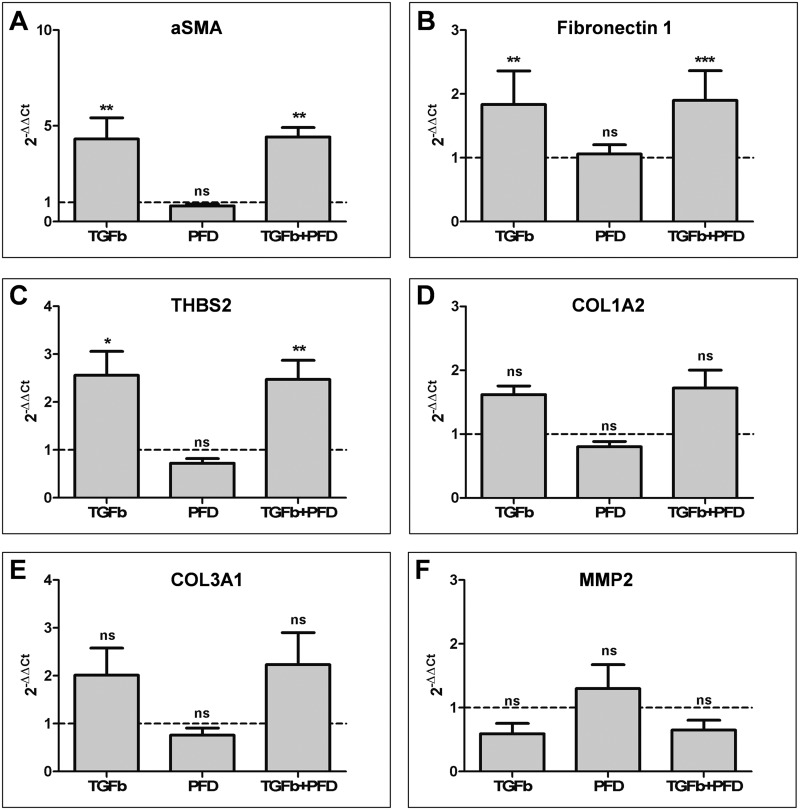
RT-PCR analysis of stimulated (TGF-β1) and suppressed (PFD) primary hOFs. Cultures of hOFs were treated with TGF-β1 [10 ng/ml], PFD [10^−3^ mol/l] or the combination of both, TGF-β1 and PFD for 48 h under serum-free culture conditions. NC (expression level of respective gene in untreated cells, dotted line) was set to 1. Data are presented as mean ± SD. The results represent the means of four independent experiments. Level of significances: *p≤0.05; **p≤0.01; ***p≤0.001. Overall, no statistically significant differences between TGF-β1 and TGF-β1+PFD groups could be observed.

In hOF cultures, almost similar results in the relative expression profiles of the abovementioned mRNAs were detected. Briefly, a 4-fold increase of α-SMA mRNA expression was observed in the presence of TGF-β1 or its combination with PFD, whereas PFD alone yielded no difference to control cultures (Fig 4A). Approximately, a 2-fold increase of the expression profiles of fibronectin (Fig 4B), THBS2 (Fig 4C), COL1A2 (Fig 4D) COL3A1 (Fig 4E) mRNAs was observed in the presence of TGF-β1 or its combination with PFD when compared to control values. In all these cases, PFD had no effect on the expression profiles of these mRNAs when compared to control hOF cultures. Similar to hTFs, also in hOFs, only a small increase in the MMP2 gene expression was observed in the presence of PFD alone, which was downregulated in the presence of TGF-β1 or TGF-β1 and PFD combination (Fig 4F).

The comparison of gene expression levels between cell cultures treated with TGF-β1 and combination of TGF-β1 and PFD revealed no statistically significant differences (Fig 4A through 4F).

Taken together, these data confirm the role of TGF-β1 as a stimulator and PFD as a suppressor of fibrosis in both fibroblast subpopulations. These effects, however, were more pronounced in hTFs.

### Immunohistochemistry

**Fig 5:**
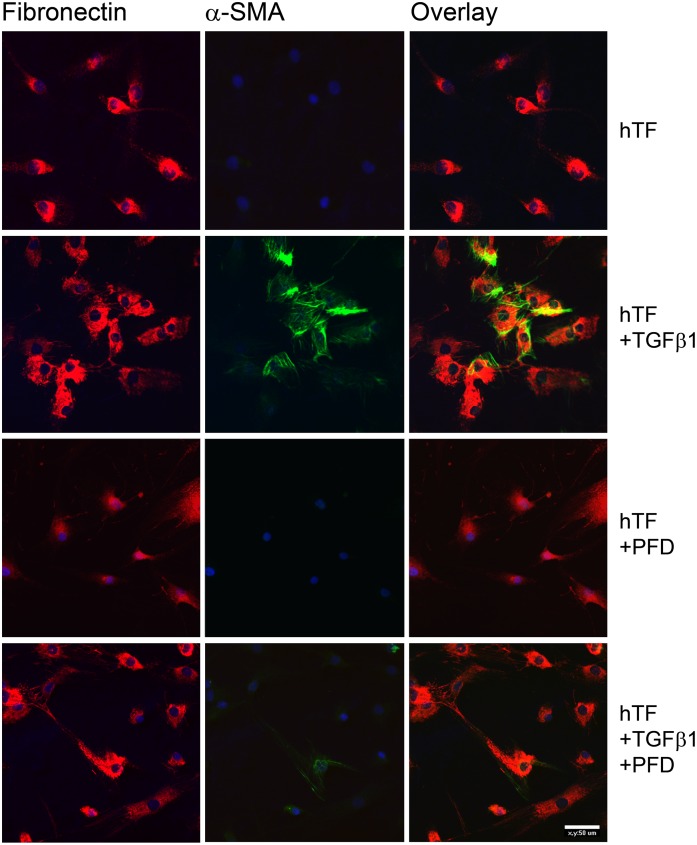
Immunocytochemical characterization of TGF-β1 induced fibronectin and α-SMA expression in primary hTFs *in vitro*. After PFA fixation, cells were incubated with primary antibodies directed against fibronectin and α-SMA. Nuclei were stained by DAPI included in the mounting medium. PFD decreased fibronectin and α-SMA expression. Experiments were carried out four times with similar results. Bar represents 50 μm.

Immunocytochemical analysis revealed TGF-β1 induced activation of primary ocular fibroblasts (hTFs) (Fig 5). In untreated hTF cell cultures or in those incubated with PFD alone, no α-smooth muscle actin (α-SMA) stress fibers were visible (Fig 5). Incubation of cells with cytokine TGF-β1 led to an expression of α-SMA, which could serve as an indicator for fibroblast transformation into fibrotic active myofibroblasts. Additionally, transformation was accompanied by an increase of fibronectin expression. A decrease of α-SMA expression could be observed in cells incubated with combination of TGF-β1 and antifibrotic agent PFD.

**Fig 6:**
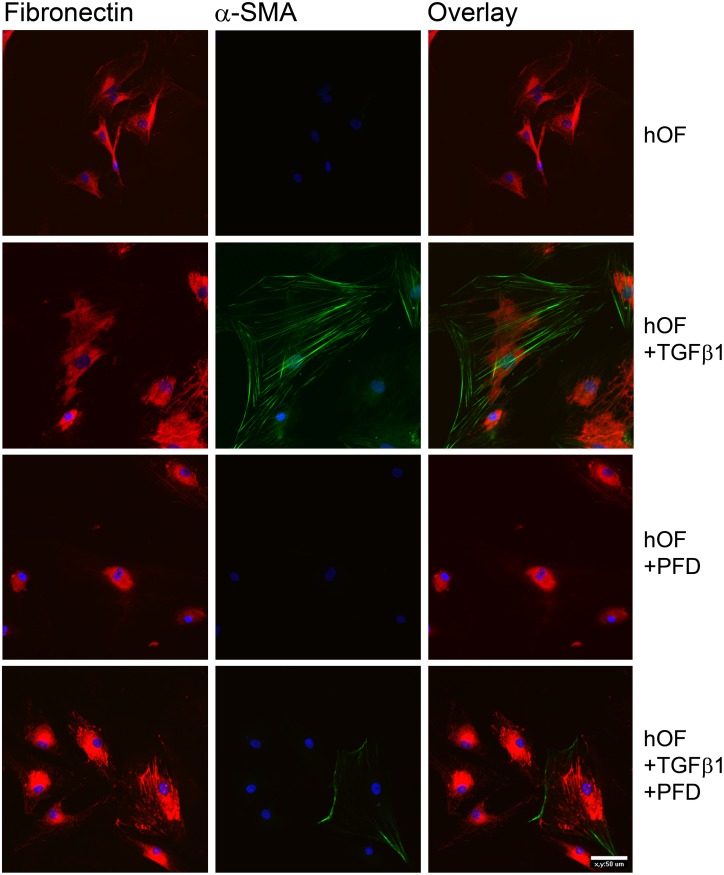
Immunocytochemical characterization of TGF-β1 induced fibronectin and α-SMA expression in primary hOFs *in vitro*. After PFA fixation, cells were incubated with primary antibodies directed against fibronectin and α-SMA. Nuclei were stained by DAPI included in the mounting medium. PFD decreased fibronectin and α-SMA expression. Experiments were carried out four times with similar results. Bar represents 50 μm.

A similar response to TGF-β1 stimulation could be detected in hOF cultures (Fig 6). While untreated control cells and cultures with PFD alone showed no expression of α-SMA stress fibers, TGF-β1 stimulation strongly increased α-SMA and fibronectin expression (Fig 6). An obvious decrease of α-SMA expression could be shown in hOF cultures with combined application of TGF-β1 and PFD (Fig 6).

Due to the fact that the transformation into myofibroblasts is associated with an increase in ECM production, we further examined the synthesis of ECM components in primary hTFs and hOFs under different culture conditions.

The immunohistochemical stainings of ECM components (collagen I, collagen III, collagen VI) under given culture conditions revealed an obvious increase in the expression in presence of TGF-β1, and only a subtle reduction in presence of PFD (data not shown).

### Western blot analysis

To compare the amounts of different ECM components, total cell lysates were obtained and analyzed by immunoblotting. As a loading control β-tubulin was analyzed, showing no differences in signal intensity and equal amounts of loaded proteins among hTFs and hOFs.

**Fig 7:**
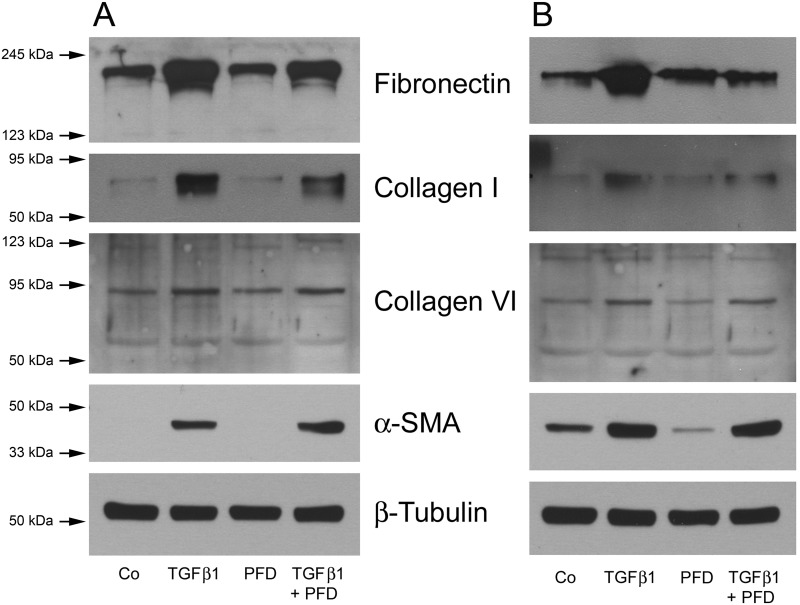
Western blot analysis of hTFs (A) and hOFs (B) under different culture conditions. Cell lysates of fibroblast subpopulations were prepared and subjected to Western blot analyses using antibodies against fibronectin, collagen I, collagen VI, α-SMA and β-tubulin as a loading control, as indicated.

**Fig 8:**
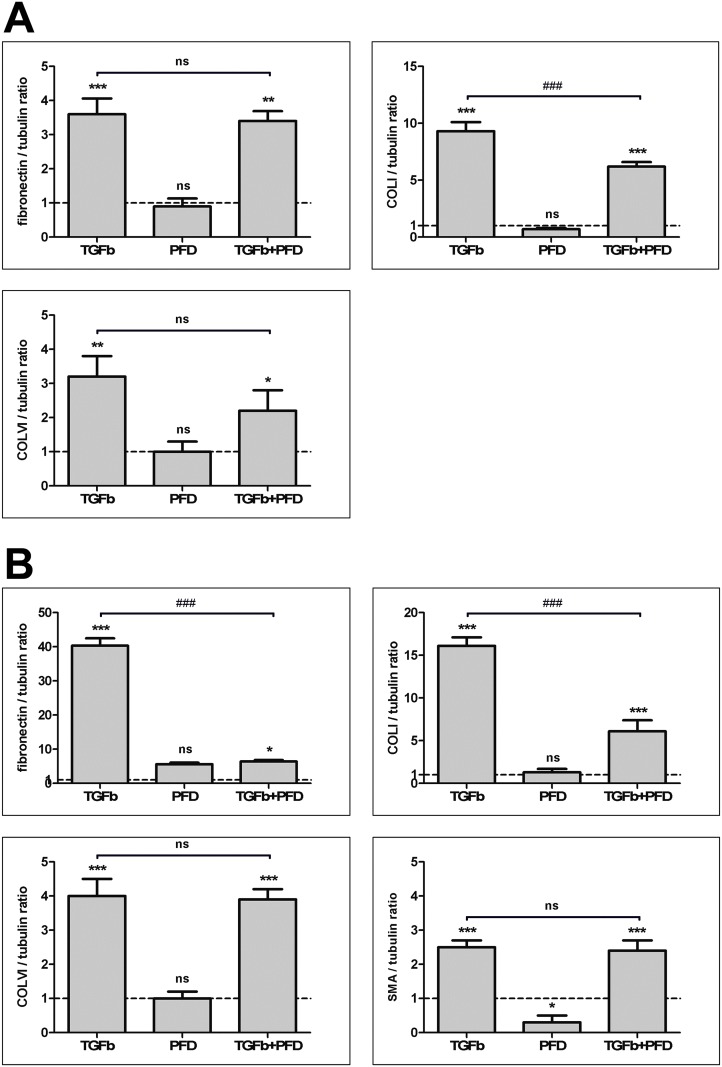
Quantification of Western blot data for hTFs (A) and hOFs (B). Each column represents the mean ± SD from three independent experiments. * indicates significances obtained by comparison of TGF-β1, PFD and TGF-β1+PFD to untreated cultures. ^#^ indicates significances obtained by comparison of TGF-β1-treated cultures and cultures with combined treatment TGF-β1+PFD. Level of significances: *^(#)^p≤0.05; **^(##)^p≤0.01; ***^(###)^p≤0.001. No quantification could be performed for α-SMA in hTFs because of the absence of the protein in control and PFD-treated samples (Fig 7).

The immunoblot analyses showed an increase in detected amounts of fibronectin, collagen I, and collagen VI in hTF cultures stimulated with TGF-β1 when compared to untreated controls (Fig 7A). When culturing hTFs with PFD alone, no changes in synthesized amounts of respective proteins could be observed. Compared to the TGF-β1 application, the combined application of TGF-β1 and PFD resulted in a decrease of collagen I and collagen VI expression (Figs 7A and 8A).

These results were also confirmed in hOF cultures (Figs 7B and 8B). Stimulation of hOFs with TGF-β1 increased the expression of fibronectin, collagen I, collagen VI and α-SMA when compared to untreated controls. When culturing hOF cultures with PFD alone, a decrease in the α-SMA expression was detected. Compared to TGF-β1 stimulated hOF cultures, the ones with combined application of TGF-β1 and PFD exhibited a statistically significant decrease of fibronectin and collagen I expression (p<0.001), as shown in Fig 8B.

## Discussion

Currently, strategies to minimize the incidence of postoperative fibrosis in glaucoma surgeries are focusing on anti-fibrotic agents, which are supposed to suppress fibrotic events in a specific and targeted way without affecting other cell types than fibroblasts.

In this context, we designed the present study to explore the effects of the antifibrotic small molecule pirfenidone (PFD), which has been previously shown to inhibit TGF-β induced protein expression and to ameliorate fibrotic processes in lung [27], kidney [28] and liver [29]. The *in vitro* experiments in our study were conducted with two different subtypes of ocular cells, namely Tenon’s and intraconal fat fibroblasts (hTFs and hOFs, respectively). As the most important cells, synthesizing ECM in the Tenon’s capsule, TFs play a key role in healing and scarring processes after glaucoma surgery at the level of the subconjunctival and episcleral tissue. The orbital fat fibroblasts, on the other hand, are of growing interest with regard to the orbital fat tissue as a possible drainage area of new generation drainage devices.

To possibly mimic the *in vivo* situation, we established fibrotic cell cultures of these ocular fibroblast subpopulations through incubation of primary cells with cytokine TGF-β1, which is considered the “master cytokine” in fibrosis [30]. It promotes fibroblast proliferation, differentiation and their transition into myofibroblasts. We could recently show that TGF-β1 was expressed in primary human ocular fibroblast subpopulations [24]. Binding of TGF-β1 to the TGF-β-receptor 1 and 2 results in a fusion of both receptors and a phosphorylation of TGF-β-receptor 1 through the TGF-β-receptor 2. This phosphorylation is transferred to the intracellular downstream-mediators SMAD2 and 3, proteins, which further transduce the signal from cell surface over cytoplasm into the nucleus, where they act as transcription factors and regulate proliferation and fibrotic genes, e.g. collagen I and fibronectin. Following incubation with TGF-β1, elevated amounts of ECM-components and α-SMA could be demonstrated in both Tenon and orbital fat fibroblasts. Furthermore, the TGF-β1 treatment resulted in up-regulation of THSB2 and downregulation of MMP2.

THSB2 belongs to a small family of secreted glycoproteins which activate the inactive, latent form of TGF-β thus enabling its interaction with cellular receptors [31]. Functionally, THSB2 plays a role in ECM turnover and is involved in physiological wound healing processes [32]. However, in fibrosis, due to the excessive ECM production, THSBs expression is increased [33]. More recently, a study examining fibrosis-related gene expression in excised capsules of failed GDDs, revealed a strong upregulation of THSB1 and THSB2 genes compared to age-matched control Tenon specimens [34].

ECM degradation under normal conditions is mediated by MMPs. One of the most important MMPs is MMP2. Down-regulation of MMP2 alters the balance of synthesis and degradation and lead to a decrease of ECM turnover with negative consequences in fibrotic matrix deposition. A down-regulation of MMP2 following TGF-β stimulation in embryonic rat fibroblasts (REF-52) was shown by Howard and colleagues [35]. Furthermore, MMP2 was clearly down-regulated in murine fibroblastic cells (NIH/3T3) and neonatal primary cardiac fibroblasts, when the cells were exposed to TGF-β [36].

These findings highlight the importance of TGF-β1 in inducing pro-fibrogenic factors (like THSB2) and suppressing proteins responsible for ECM homeostasis (like MMP2). Expectedly, upon TGF-β1 incubation the cells exhibited also an increase in cell proliferation, compared to untreated cultures (6- and 3-fold for hTFs and hOFs, respectively).

In the next step we examined the antiproliferative and antifibrotic effects of PFD, both in control and TGF-β1 treated “fibrotic” cell cultures. The broad antifibrotic spectrum of PFD, including TGF-β1-suppression was demonstrated in numerous *in vitro* and *in vivo* studies [37]. PFD was also shown to inhibit cell proliferation, myofibroblast differentiation, collagen contractility and migratory ability of cardiac fibroblasts [38]. Similar effects were observed in hepatic and renal fibroblasts following PFD treatment [39,40]. Also in human Tenon fibroblast cell cultures PFD inhibited cell proliferation in a concentration- and time-dependent manner by arresting the cells in the G1 phase of the cell cycle [41]. The same study also demonstrated for the first time the antifibrotic effects of PFD on ocular fibroblasts, as shown by reduced cell motility, collagen contraction and down regulation of TGF-β1 and 2 gene transcription along with decreased expression of respective proteins. In good agreement with existing literature, our results also demonstrate that PFD inhibits cell proliferation and slightly inhibits ECM production and cell differentiation into myofibroblasts in unstimulated cell cultures. We can rule out any possibility, that antiproliferative effects of PFD are mediated by drug toxicity as viability assays showed that PFD is not cytotoxic in the given concentration.

All above mentioned literature findings on PFD have been obtained from *in vitro* experiments using unstimulated cells. So far, there is only one study, which has demonstrated the antifibrotic effects of PFD in a “fibrotic” cell culture of ocular fibroblasts [42]. This culture was induced by exposure to interleukine-1β (IL-1β), while TGF-β and other cytokines failed to stimulate the cells. In contrast, here, we could successfully establish a “fibrotic” culture through exposure of Tenon and orbital fat fibroblasts to TGF-β1.

Surprisingly, in such cultures the antiproliferative and antifibrotic effects of PFD were less pronounced. Cells, simultaneously incubated with TGF-β1 and PFD were almost completely comparable with “fibrotic” ones (incubated with TGF-β1 alone) by mean of cell proliferation and production of ECM-components. The only markers, which were reduced compared to fibrotic cultures were the hallmark of myofibroblasts α-SMA and the ECM component fibronectin.

In clinical terms, the above mentioned results might implicate, that once fibrosis has developed, PFD would not be able to make the scarring processes reversible.

When translating these observations to the *in vivo* situation, one possibility is to apply PFD either postoperatively in form of eye drops after filtration surgery or GDD implantation, or incorporated in GDD for a sustained local drug delivery. In both cases, PFD will be present on the target site before fibrosis occurs, which means that this drug can play a preventive role in the development of fibrotic events. A randomized, controlled masked-observer rabbit study has shown that a postoperative use of PFD was associated with less scarring and improved trabeculectomy bleb survival [43].

In conclusion, we report about the differential expression of several genes and proteins in TGF-β1 stimulated ocular fibroblasts, compared with normal untreated cells. The results of the present study suggest that PFD is able to exert antifibrotic effects on untreated cells, while being less effective in TGF-β1-stimulated “fibrotic” cultures.

Combined with the results of previous *in vitro* studies performed on ocular fibroblasts, we propose that PFD may be a promising candidate for the treatment of fibrosis following glaucoma filtration surgery or GDD implantation.
